# Allergen Immunotherapy: Current and Future Trends

**DOI:** 10.3390/cells11020212

**Published:** 2022-01-08

**Authors:** Gandhi F. Pavón-Romero, Maria Itzel Parra-Vargas, Fernando Ramírez-Jiménez, Esmeralda Melgoza-Ruiz, Nancy H. Serrano-Pérez, Luis M. Teran

**Affiliations:** Department of Immunogenetics and Allergy, Instituto Nacional de Enfermedades Respiratorias Ismael Cosío Villegas, Mexico City 14080, Mexico; gandhifernando@hotmail.com (G.F.P.-R.); itzelparra7777@hotmail.com (M.I.P.-V.); dr_frj@yahoo.com.mx (F.R.-J.); esme.melgoza.ruiz@gmail.com (E.M.-R.); nan.serrano.p@hotmail.com (N.H.S.-P.)

**Keywords:** hypoallergenic immunotherapy, allergen immunotherapy, recombinants, adjuvants

## Abstract

Allergen immunotherapy (AIT) is the sole disease-modifying treatment for allergic rhinitis; it prevents rhinitis from progressing to asthma and lowers medication use. AIT against mites, insect venom, and certain kinds of pollen is effective. The mechanism of action of AIT is based on inducing immunological tolerance characterized by increased IL-10, TGF-β, and IgG4 levels and Treg cell counts. However, AIT requires prolonged schemes of administration and is sometimes associated with adverse reactions. Over the last decade, novel forms of AIT have been developed, focused on better allergen identification, structural modifications to preserve epitopes for B or T cells, post-traductional alteration through chemical processes, and the addition of adjuvants. These modified allergens induce clinical-immunological effects similar to those mentioned above, increasing the tolerance to other related allergens but with fewer side effects. Clinical studies have shown that molecular AIT is efficient in treating grass and birch allergies. This article reviews the possibility of a new AIT to improve the treatment of allergic illness.

## 1. Introduction

Allergen immunotherapy (AIT) originated in the early twentieth century [1]. In 1954, the first controlled clinical trial was developed, and AIT improved the symptoms in the group receiving the pollen extract compared with the control group [2]. AIT is recommended for the treatment of allergic rhinitis (AR) and asthma by many medical organizations, based on controlled clinical trials and meta-analyses [3,4,5]. Many of these beneficial effects are due to AIT being the unique therapy able to induce allergen long-term tolerance after discontinuation. The administration of AIT for three years produces persistent clinical-immunological changes for at least two years [6,7].

**Figure 1:**
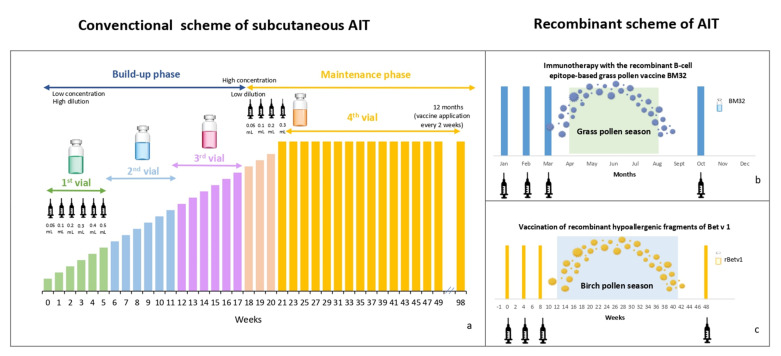
Schemes of allergen immunotherapy. (**a**) Classical subcutaneous immunotherapy scheme consisting of build-up and maintenance phase. (**b**) Recombinant scheme with grass (**c**) Recombinant scheme with birch allergen.

However, the conventional schemes used in AIT are prolonged. Subcutaneous immunotherapy (SCIT) comprises a build-up phase (in which the allergen concentration increases gradually) and a maintenance phase (in which the projected dose is applied), which must be administered for at least three years (Figure 1a). Additionally, the development of adverse reactions is associated with first dosages, leading to treatment abandonment [8,9]. Likewise, AIT has not shown clinical benefits with all allergens.

New methods and novel molecules have been developed to improve AIT for the last 30 years. Currently, allergoids, recombinant allergens based on specific epitopes or joined to immunological adjuvants—also known as hypoallergenic immunotherapy—even applied by new routes, constitute new variants of this therapeutic. The present review describes the best advances reported concerning each of the AIT areas.

## 2. Mechanisms of Action of AIT

**Figure 2:**
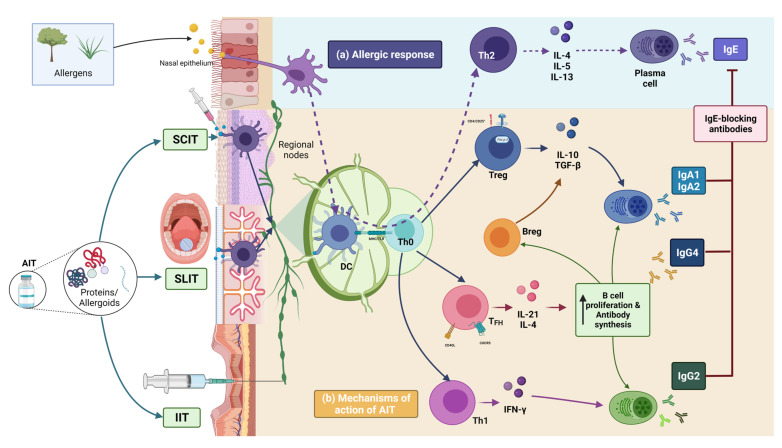
Mechanism of allergen-specific immunotherapy. (**a**) The allergic response begins with the allergen being endocytosed by the dendritic cells of the airway epithelium (DC). Subsequently, DC goes to local secondary lymphoid organs (lymph nodes) where the antigen presentation to Th0 happens. Th0 differentiate to Th2 and synthesize its interleukin profile to allow the production of IgE-type specific allergen antibodies. (**b**) Subcutaneous, sublingual, and intralymphatic immunotherapies (SCIT, SLIT, IIT) provides the antigen (peptide, recombinant, or protein complex) and induces T naive cells differentiation into different types, which synthesizes their interleukin profiles as Th1 (IFN-γ) or Treg Foxp3^+^ (IL-10 and TGF-β). TFH CXCR5^+^ (IL-21 and IL-4) profiles co-helping plasmatic cells to produce IgA1, IgA2, and IgG4 antibodies block against IgE-allergen specific.

The purpose of the AIT is to modulate the physiopathology mechanisms of allergy. The allergic response begins with the allergen being endocytosed by the dendritic cells of the airway epithelium. Subsequently, these cells go to local secondary lymphoid organs where the antigen presentation to Th0 happens. Th0 differentiate to Th2 and synthesize its interleukin profile (IL-4, IL-5, and IL-13) to allow the production of IgE-type specific allergen antibodies (Figure 2a).

The mechanism of AIT includes an increase in the number of T regulatory cells (Treg) CD4^+^CD25^+^ [10] and IgG4 levels [11]. Treg cells produce TGF-β [12], IFN-γ, and IL-10, which are essential for AIT’s immunomodulatory activities [13,14]. For example, IL-10 inhibits the histamine release by mast cells mediated by its IgE-dependent activation [15] and a decrease in eosinophil cationic protein release [16]. Likewise, the combination with IL-4/IL-13 allows the isotype change to IgG4 instead of IgE in plasma cells [17,18]. IFN-γ inhibits the synthesis of Th2 interleukins as IL-4, IL-5, and IL-13. AIT is also linked to a reduction in antigen-specific T cell clones and IgA2 production. Recently, AIT increased the differentiation of T follicular reg cells-TFH (CXCR5^+^ Foxp3^+^) and B regulatory cells—Breg [19]. In the first case, TFH synthesizes the IL-21 and IL-4 necessary for B cell proliferation and antibody synthesis [20]. B cells secrete IL-10/TGF-β, both of which help with immune modulation [21]. These mechanisms are reference points for other new routes, molecules, and adjuvants used in AIT. However, some effects have specific routes (Figure 2b).

SCIT increases the Treg profile and IgG-mediated blockade of the binding of IgE and allergens to plasma cells after twelve months [17]. Interestingly, the titers of the blocking antibodies induced by AIT increase approximately 30 times more than by sublingual immunotherapy (SLIT) in less time (6–10 weeks) [22]. In this context, nasal mucosa biopsies from grass-sensitive patients increased the levels of IL-10 mRNA after two years of SCIT treatment [23].

Although the mechanism of action of SCIT has been studied further, the SLIT mechanism has similarities with the one already explained [24,25]. Langerhans cells in the oral mucosa are essential for the antigen presentation and the activation of T lymphocytes. The Toll-like receptor (TLR) TLR4 activation in these cells increases the synthesis of IL-10, TGF-β, IFN-γ, and IL-2 and Foxp3^+^ expression in Treg cells from both oral epithelium biopsies and peripheral blood [18,26]. Ihara F. reported that the SLIT applied for 52 weeks in house dust mite (HDM)-sensitive AR patients also decreases the Th2 cells [27], as well as in ryegrass pollen-sensitive patients in six months [12,27]. However, the synthesis of blocking IgG4 antibodies occurs over a longer time (~24 weeks) than that using SCIT (6–10 weeks) [28], maintaining the increase in IgG^+^ memory B cells 1–3 years after treatment [29,30].

There is evidence that the rupture of the skin barrier facilitates the penetration of allergens into the epidermis, causing Langerhans cells to catch allergens and travel to regional lymph nodes, with the subsequent stimulation and differentiation of T lymphocytes toward a Th2 profile [31]. This mechanism is the basis of percutaneous AIT, which causes a more potent delayed T-cell-mediated immune response than SCIT and SLIT. Unfortunately, the synthesis of IgG4 is less than that using the routes mentioned above [32,33].

Additionally, an inguinal node AIT injection with recombinants (phospholipase A2 and Fel d 1) increased IgG levels 10 times more in a shorter period (two weeks) than SCIT with a dose 100 times lower. These effects are probably due to the allergens reaching the lymph nodes directly compared with SCIT or other routes. Interestingly, this administration route is the only one that produces both IgG2 and IgG4. Similarly to the other routes, intralymphatic immunotherapy induces increased IL-10, IFN-γ, and IL-4 levels but in a shorter time than SCIT [34]. The advantages of this route are that it is painless, and the tolerance against allergens is achieved in less time (four months) and with more durability. However, a disadvantage of this approach is that it should be used under ultrasound guidance [35].

## 3. Efficacy of Allergen Immunotherapy

The benefits of AIT are not the same for each allergen responsible for sensitization and the different allergic diseases. For example, SCIT or SLIT is strongly recommended for seasonal AR induced by pollen, while SLIT is recommended for HDM in mild asthma but not for all allergens [36,37,38,39], and there is limited evidence for allergic respiratory disease caused by fungal spores [40,41]. The meta-analyses are considered the highest level of evidence The SCIT meta-analyses for seasonal AR showed an improvement in symptoms (SMD, −0.73; *p* < 0.0001; I^2^ = 63.21%) and medication scores (SMD, −0.57; *p* < 0.0001; I^2^ = 64.02%). Interestingly, this evidence is mainly derived from articles on AIT for grass pollen. However, SCIT against HDM also showed similar results (For the symptoms: SMD, −2.17; *p* = 0.001; I^2^ = 96%%/For the medication score: (SMD, −1.17; *p* = 0.03; I^2^ = 86%). Concerning SLIT, the most up-to-date version of the Cochrane review reports described a reduction in the outcomes mentioned above, primarily for grasses (For the symptoms: SMD, −0.49; *p* < 0.00001; I^2^ = 81%/For the medication score: SMD, −0.32; *p* = 0.00035; I^2^ = 50%), and other robust reports concluded the same for HDM (For the symptoms: SMD, −0.95; *p* < 0.00001; I^2^ = 92%/For the medication score: SMD, −1.88; *p* < 0.00001; I^2^ = 95%) [38,42]. Some reports have found similar levels of efficacy using both routes, even comparing different forms of SLIT (drops and tablets) [43,44]. In relation to SLIT and asthma, a recent meta-analysis could not draw clinically useful conclusions due to the non-validated scores and limited evidence for relevant outcomes such as asthma exacerbations [37]. For other allergens, there is scarce high-quality information. However, evidence supports a clinical improvement in SCIT and SLIT for epitheliums in clinical outcomes such as ocular, nasal, or asthma symptoms, peak expiratory flow rate, and medication scores [45].

Notably, some meta-analyses, particularly those using SLIT, are controversial because of the heterogeneity of the few included trials, different presentations, and doses of the extracts used, and/or of the use of non-validated scales of symptoms and medication scores, limiting the provision of clear clinical conclusions. Additionally, heterogeneity exists in the different clinical trials included in the meta-analyses. Throughout history, an attempt has been made to improve the effectiveness criteria and propose a consensus on the duration of SCIT and SLIT [46]. Furthermore, one of the most interesting properties of AIT is that it provides benefits for many years after the therapy schemes have been concluded. Patients have a reduction in medication and the percentage of eosinophils, as well as an increase in the threshold to the response to methacholine four years after finishing the AIT, according to prospective studies evaluating SLIT regimens administered for at least three years, and even these effects are more prolonged with schemes applied for a longer time [47,48]. In a similar context, the application of a complete AIT scheme for mites avoids the development of new sensitizations in 75% of patients at least three years after its conclusion [49,50].

Regardless of these scores, some previously discussed interleukins (IL-10, TGF-β), antibodies titers (IgG4) [50], IgE [51], specific IgE/total IgE [52], and cell lines (Treg cells, B regs and DC) have been used as biomarkers [53]. Although the modification of other types of lymphocytes and immune cells have also been described. For example, AIT for grasses increase the expression of the transcriptional factor of DCreg (C1QA, FcεRIIIA, FTL) and reduced that of DC2 (C1QA, FcεRIIIA, FTL,); in a similar way, it diminished the expression of CD63/CD203c in basophils, which correlates with the medical score and is considered as a biomarker of efficacy [52,54].

SCIT and SLIT are generally well tolerated. However, as another therapeutic approach, they are not exempt from the development of adverse reactions. The risk of a systemic reaction is more frequent with SCIT than SLIT. A systemic response to AIT injection is documented in approximately 2% of patients, and the mortality related to a SCIT injection is higher in non-controlled asthma patients in the build-up phase and maintenance [8,48]. Low-risk fatal reactions occur in 1 per 2.5 million injections, although this rate has decreased in recent years [55,56]. SLIT is considered the safest route, even in asthma patients [37].

Recently, attempts have been made to improve the adaptability and success of immunotherapy using monoclonal antibodies, particularly with the anti-IgE monoclonal antibody (Omalizumab). Omalizumab in immunotherapy is an off-label treatment. However, clinical trials have shown that the use of Omalizumab in rapid regimens of AIT, such as rush, reduces the adverse reactions attributed to immunotherapy [57]. Another trial showed benefits in the symptom control of seasonal rhinoconjunctivitis and asthma when used before or during immunotherapy schemes [58,59]. However, it will unlikely be approved as a general indication because of its high cost and limited and probably temporary clinical benefits.

## 4. Allergoids

Allergoids are chemically modified allergens via polymerization with glutaraldehyde or formaldehyde. This modification gives them better immunogenicity features [60] because they react with primary amino groups in the polypeptide chain of the allergen, producing intramolecular and intermolecular cross-linked polymers of high molecular weight allergens [61]. The conformational epitopes of IgE are destroyed, while the linear epitopes of T cells are not affected [62]. This structure allows its administration in high doses during a short-term accumulation phase [63].

Since 1992, diverse allergoids have been used; for example, a mixture of six grass allergens modified with formaldehyde and co-precipitated with aluminum, known as Allergovit^®^, has been applied in a phase-IIIb study by the subcutaneous route in AR patients [64]. In a phase-III clinical trial, allergoids enhanced the levels of subclass IgG (IgG2 and IgG4) antibodies for grasses [65], decreased basophil activity [57], and relieved allergic symptoms after one year of treatment, doubling this effect in the second year in a phase-III study [65]. However, similar mechanisms have also been shown with allergoids derived exclusively from one grass (*Phleum pretense*) in a phase-II study. This activity is attributed to the high sequence identity of group 1 and group 5, ~90% and >55%, respectively, among members of the Poaceae family [66]. Instead, Pfaar O. evaluated other glutaraldehyde and formaldehyde (Acaroid^®^) allergoids for HDM. They identified that doses ≥20,000 UA and 18,000 TU improve the threshold of symptoms in nasal provocation tests and increase blocking antibody levels, respectively [67,68]. This molecular result was also shown with allergoids of birch (Allergovit^®^ Birch phase-II study and PURETHAL^®^ phase-IV study) [69,70].

In the same context, the carbamylation of the lysine groups has been used to develop allergoids of low molecular weight that can be absorbed easily by the mucosa as the monomeric allergoid of group five of *Phleum pratense* (Grazax^®^ 75,000 standardized quality units) [71]. This protein, applied sublingually, reduced the need for antihistaminic drugs during the pollination season, in addition to the clinical effects mentioned with the other molecule [72]. Additionally, this allergoid maintained its clinical benefits after termination for at least two years [73]. The allergoid LAIS^®^, a mixture of extracts from group-1 mites, was another carbamylated chemical employed in a phase-II research. LAIS^®^ applied by SLIT at doses of 3000 UA over one year reduced the IL-4 and augmented IFN-γ levels. Additionally, it improved rhinitis severity and reduced drug intake [74]. In the same context, Hüser C. evaluated other similar allergoids but applied them for 12 weeks and noted that 2000 UA/day decreased the symptoms in conjunctival provocation tests [75].

Concerning its safety, the patients treated with Allergovit^®^ for grass allergy in phase-II studies developed mild reactions [76,77], even when applied during the pollination season [64], suggesting that utilizing allergoids in rapid treatment schemes may allow patients to achieve clinical and immunological tolerance more quickly, as well as enhance the adherence to AIT [77].

## 5. Adjuvants

An adjuvant is a molecule that enhances immune responses by interacting with antigens physically or chemically [78] and has been traditionally classified as first-generation (aluminum, microcrystalline tyrosine and calcium phosphate) and second-generation (Toll-like receptors—TLR). Additionally, other adjuvants have been used with promising results, such as liposomes and virus-like particles. (Table 1).

### 5.1. Aluminum

Aluminum-containing adjuvants (ACDs) have been used widely for AIT [79,80]. ACDs have adsorbent properties, and their administration causes the local deposition of antigens, which are released slowly [81]. The injection of an ACD intramuscularly induces uric acid release in the muscle; both molecules stimulate NOD-like receptors (NLRs), specifically NALP3, an essential component of the inflammasome, along with Pycard and caspase-1, subsequently promoting the synthesis of inflammatory interleukins (IL-1, IL-18, and IL-33) in monocytes [82,83]. IL-1 primarily induces a Th2 response, which is necessary to synthesize IgG subclass antibodies, and this last effect is not fully understood [84]. An increase in the IgG subclass has been shown in AIT, which comprises an ACD and fragments of Bet v 1 (aa 1–74 and aa 75–160) [85]. This adjuvant’s clinical efficacy has been proven: Corrigan C. evaluated ACD-containing Allergovit^®^ for grasses, and this vaccine reduced the symptoms and drug requirements [65]. Furthermore, a phase-IIb study reported that an ACD-containing synthetic contiguous overlapping peptide (COP) vaccine for Bet v1 improves the quality of life of AR patients [86]. ACDs have been integrated into many glutaraldehyde allergoids, and patients who receive these treatments develop mild reactions [87]. However, some reports have indicated that ACDs induce granulomas in the skin and severe reactions inherent to AIT and, in some cases, are associated with neurologic diseases [88,89].

### 5.2. Microcrystalline Tyrosine (MCT)

L-tyrosine is an amino acid that delays the bioavailability of allergenic materials [90]. Since first reported in the 1980s, the adsorption of allergenic molecules to L-tyrosine has enhanced the induction of IgG [91]. MCT has the property of being rapidly released and metabolized, unlike aluminum, which can be found at the sites of application in murine models of AIT and is associated with the development of granulomas [92]. MCT has not shown toxic properties, although it is contraindicated in tyrosine metabolism disorders [90]. Currently, MCT is patented as an adjuvant for use in immunotherapy [93] and is integrated into glutaraldehyde allergoids for *Dermatophagoides* to help reduce allergic symptoms and diminish the use of relief drugs [94,95,96,97].

### 5.3. Calcium Phosphate (CaP)

Calcium salts such as CaP [98] and MCT share physical properties and immunological mechanisms. Allergens are adsorbed by CaP in the shape of microcrystals at the injection site, releasing the antigen slowly in encapsulated particles toward the APC [99]. CaP plus five-grass pollen extract improves nasal symptoms and increases the noise reactivity in nasal challenges as well as the IgG4 levels [100]. CaP has been used as an alternative to ACD [101]. However, patients treated with CaP developed double subcutaneous local reactions [102], but this effect disappears when it is applied intramuscularly [101].

### 5.4. Toll-like Receptors (TLR)

The main evidence of their potential usefulness in AIT comes from in vitro studies. For example, resquimod, an agonist of TLR8/9, increases the synthesis of IFN-γ (suppressor of the allergic inflammatory response) in PMNC derived from AR patients sensitized to palm pollen [103]. TLR2/6 can be stimulated by a lipopeptide derived from Mycoplasma, known as Macrophage Activating Lipopeptide of 2 kDa, which decreases the Th2 profile and eosinophil counts in bronchoalveolar lavage, but has no effect on T-reg cells in mice sensitized to *Phleum pretense* [104]. In a mouse allergy model, fusion proteins containing recombinant flagellin A (TLR5 agonist) and Bet v 1 (rFlaA:Betv1) decreased the Th2 responses and avoided allergic sensitization compared to rBet v 1 [105]. Monophosphoryl lipid A (MPL) is a TLR4 agonist, which is a lipopolysaccharide of the cell wall of *Salmonella minnesota* that stimulates the production of IFN-γ and IL-12, but does not promote IL-5 synthesis [106]. In a phase-I/IIa study, patients treated with MPL and mixed-grass pollen developed an increase in IgG with lower IgE levels and low nasal reactivity following a grass challenge [107]. Similarly, Worm M. evaluated its role with an allergoid of birch and showed that the administration of MPL-birch reduced basophil activation more than 100-fold compared with native allergens [108].

In the same context, MPL integrated into the Pollinex Quattro allergoid vaccine for grass allergy, an alternative ultrashort (four pre-seasonal injections) of SCIT, induces increases in CD4^+^, CD25^+^, Foxp3^+^, and IgG antibodies but not IgE; in addition to improving the allergic symptoms [109,110,111], even after increasing the cumulative dose, its safety is not compromised [112]. Regarding the long-lasting effect of MPL, patients who received MPL-Parietaria (a scheme of four injections before the pollen season for three years) applied using the SCIT route showed clinical improvement up to five years after treatment discontinuation [113]. Similar results were obtained in patients with the cessation of Pollinex Quattro after three years [114]. In the case of TLR intracellular agonists such as cytosine-phosphate-guanosine (CpG) to stimulate TLR9, CpG and a recombinant *Chenopodium album* increased IL-10 and IFN-γ and reduced IL-4. B cells from patients sensitive to cedar also reduced IL-5 and IL-13 [115,116]. New agonists of TLR continue to be evaluated, such as AZD8848-TLR7, and showed an improved lung function in asthma patients in a phase-II study [117]. These findings suggest that TLR regulation may be a promising therapeutic approach in allergic diseases.

### 5.5. Liposomes

Liposomes are spheres or vesicles integrated by lipids such as cholesterol and/or phosphatidylcholine that allow the encapsulation of allergens [118]. Liposomes constituted by cationic lipids allow for a better interaction with DCs and are released, processed, and subsequently presented to T cells [119,120]. In a mouse model of food allergy, liposomes containing synthetic neoglycolipids such as mannotriose and dipalmito-ylphosphatidylcholine activate T CD8^+^, T CD4^+^, CD25^+^, and Foxp3^+^ cells, inhibiting the antibodies and alleviating the allergic symptoms [121]. In the same context, in an AIT for cockroaches, recombinants of arginine kinase Per a 9 encapsulated in this vehicle and applied nasally to cockroach-allergic mice reduced the inflammatory response mediated by the Th2 profile and increased the expression of IL-12, IFN-γ, and IL-10 [120]. Interestingly, when combined with Tregitopes, the liposomes augmented IL-10 and TGF-β but decreased lung inflammation and airway remodeling [118]. Der p 1 coated in liposomes was administered for one year in asthma patients; after treatment, the patients showed reduced symptoms and an increased threshold to methacholine challenges, as well as eosinophilic inflammation, compared with the control group [122,123].

### 5.6. Virus-like Particles (VLPs)

VLPs are produced from viral capsid proteins and have the potential to activate the immune system through innate mechanisms (PAMPs) that are not dependent on T cells [124]. The main reports of VLP efficacy come from animal models. For example, Fel d 1 incorporated into VLPs derived from a cucumber mosaic virus and applied to an allergic model animal induces a specific IgG response [125]. In humans, CYT003-QbG10 VLPs (TLR9 agonists arranged into VLPs) improve the AR quality of life, asthma symptoms, and lung function [126,127]. Extensive reviews on the role of adjuvants in AIT have already been published [128,129]

## 6. Peptides and Recombinants

The specificity of the protective antibodies targets the epitopes, not complete antigen molecules. Epitopes are linear segments (~20 aa) of antigen from molecules located in the major histocompatibility complex (MHC) after the APC process during the specific immune response, which induces the synthesis of antibodies or T cell clones [130]. Many products used in hypoallergenic immunotherapy include T cell epitopes. These are classified into short (~20 aa) and overlapping peptides (long peptides), which are a complete allergen sequence with overlaps, ensuring the presence of all possible T-cell epitopes. In both cases, the lack of conformational structures cannot activate IgE-dependent mechanisms [131,132].

**Figure 3:**
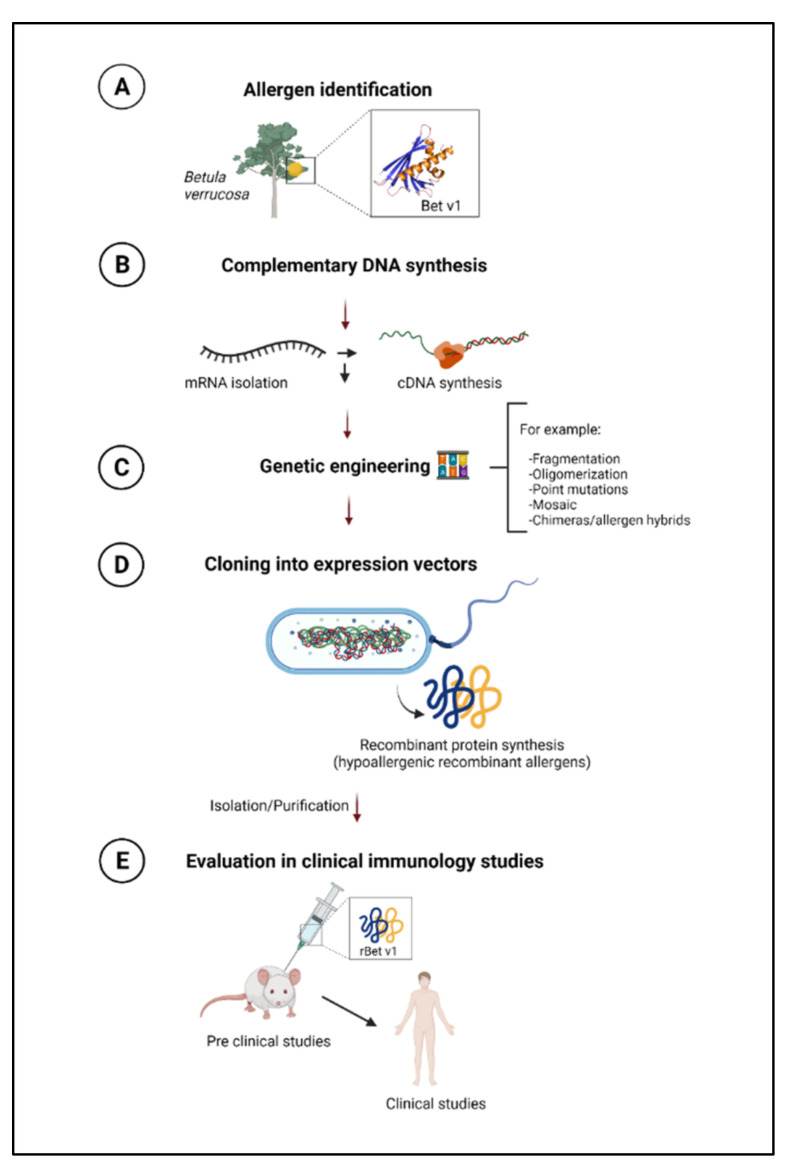
Method for synthesizing recombinant allergens. (**A**) Identification of the amino acid sequence of the proteins associated with allergic symptoms (allergen); (**B**) Isolation of the messenger RNA through the use of the genetic code and creation of the successive complementary DNA (cDNA) with the reverse transcriptase enzyme constituting the specific gene for this protein; (**C**) Insertion of the cDNA sequence into the bacterial genetic material (*Escherichia coli*) and polymerization of the recombinant cDNA, (**D**) Insertion of the recombinant cDNA into the host microorganism with the subsequent synthesis of hypoallergenic recombinant allergens, (**E**) Evaluation in clinical-immunology studies.

In a similar context, recombinant hypoallergens have been designed from native allergens. For example, these molecules show conformational changes in their IgE-binding epitopes, a quality that reduces their immunoallergenicity. The use of recombinant allergens makes it possible to have homogeneous and well-defined standardized products in adequate quantities without undesirable materials that impair the stability of the allergen and their efficacy. The most commonly used technique for recombinant synthesis includes complementary DNA (cDNA), previous identification of allergen (Figure 3A,B). cDNA is a molecule copied from an mRNA molecule by reverse transcriptase and lacks the introns and regulatory sequences present in genomic DNA (Figure 3C). The insertion of a cDNA sequence into the bacterial genome (*Escherichia coli*) encodes a recombinant protein allergen (Figure 3D). Finally, the recombinant allergen must be validated as another new drug or molecule (Figure 3E). Usually, researchers evaluate the binding to specific IgE, the release of preformed products of basophils or mast cells, the type of interleukin profile induced, the stimulation of cellular clones, and the synthesis of blocking antibodies [133]. Recombinants have been used for synthesized B-cell epitopes for therapeutic uses [134].

However, they can be produced by other methods, such as oligomerization, point mutation, fragmentation, mosaic, and allergen hybrids [135]. Recently, the in silico models have been used for developing AIT for *Dermathophagoides*. For example, a recombinant allergen of grasses developed by point mutations—the substitution of amino acids located at the calcium-binding site—produces a decrease in the negative charge of the mutant, endowing it with greater flexibility, which is important in the development of the side effects [136]. Another hybrid recombinant of ambrosia pectate lyase (rAmb a 1, residues 1174–397) and mugwort (rArt v 6, residues 173–396), as well as a hybrid of both, have been investigated. The recombinant proteins and chimera did not cause the recognition of IgE in patients sensitive to these weeds or the degranulation of preformed mediators or cytokines of the Th2 profile [137]. Additionally, a fusion protein (rFlaA: Art1hyp), integrated by flagellin A (TLR5 agonist) and hypoallergenic mugwort (Art v1- change in cysteines for serines, altering the epitope for IgE), stimulates the synthesis of subclasses of IgG but not of IgE [138].

Computational vaccine design (in silico design) entails the use of computational tools to map epitopes, select antigens, and develop immunogens [139]; through the design of new molecules and their model in 3D (I-TASSER software), it is validated (ProSA-web) and subsequently synthesized (GenScript). The hybrid proteins synthesized can incorporate many allergen determinants of the same or different species and have been evaluated mainly in allergic murine models for mites. For example, Ferreira F. created two hybrid proteins from *Blomia tropicalis* fragments (Blot t 5 and Blot t 21). Another hybrid protein is Dpx4, which contains antigenic regions of allergens from *Dermathophagoides pteronyssius* (Der p 1, Der p 2, Der p 7, and Der p 10). MAVAC-BD-2 is the first molecule to contain epitopes from *Dermatophagoides* sp. (Blo t 5, Blo t 8, Blo t 10/Der p 1, Der p 2, Der p 7, Der p 8). This protein reduced IgE to Blot 5 and Der p 2 by 20%, approximately, while boosting IgG. Concerning the limits of these products, it is probable that the addition of aggregates could impact protein stability [140,141,142]. The clinical-immune efficacy of some recombinants is better when they are integrated with adjuvants such as MPL or aluminum. For example, MPL coupled to *Phleum pratense* recombinants (rPhl p 1, rPhl p 2, rPhl p 5) induces a potent humoral response mediated by IgG and IgM and reduces the histamine release from basophils, improving the allergic symptoms [143]. The second adjuvant improved AR patients’ quality of life and increased IgG1 titers in the first year after the therapeutic scheme ended. However, a higher increase in IgG4 was observed until the second year [144]. Peptides and recombinants have been tested in phase-I/II clinical trials to treat cat and pollen allergies.

## 7. Clinical-Immune Efficacy of Recombinant Allergens

### 7.1. Cat

Fel d 1 is the most common cat allergen. Fel d 1 hypoallergenicity can be synthesized by introducing duplications of T cell epitopes (DTE). In a murine cat allergy model, a type of recombinant DTE III induced high IgG2 levels. In mice, IgG can reduce skin reactivity and improve airway hyperreactivity by blocking the binding of patients’ IgE to rFel d 1 [145]. AIT for Fel d 1 has been tested in vaccines based on T cell epitope peptides (SPIRES), which are short allergen peptides that make up the allergen’s primary T cell epitopes, and MHC II has been used to construct immune-therapeutic mechanisms [146]. Allervax cats (cat peptide for AIT) showed clinical benefits; however, they had late adverse reactions in clinical phases [147]. Conversely, in phase-II and -III studies, a Cat PAD (also known as ToleroMune Cat) has also shown a reduction in rhinoconjunctivitis symptoms and safety in cat-allergic patients using four intradermal doses of 6 nmol [148,149], decreasing the CRTh2 expression but not altering the number of Fel d 1-TCD4+ cells [150].

In a phase-I research, rFel d 1 was also fused to the HIV-derived translocation peptide (TAT), mediating the cytoplasmic uptake of extracellular proteins and the truncated human invariant chain (MALT-Fel d 1), which was administered intralymphatically in a scheme of three dosages. MALT-Fel d 1 improved the symptoms during the nasal challenge and increased the IgG4 and IL-10 levels [151]; this humoral response was greater than that of another IgG subclass, which increased after the first month of treatment. Interestingly, rIgG4 for cat allergy has been evaluated in cat-allergic patients in a phase-Ib study, demonstrating its ability to increase the IgG/IgE ratio and decrease the clinical symptoms in nasal provocation, with similar results in a scheme of eight days. These data suggest that passive immunization can treat allergies using allergen-specific IgG antibodies [152].

### 7.2. Birch

rBet v 1 is one of the first molecules evaluated as allergen immunotherapy [138]. Niederberger V. realized in 2004 a phase-II study and administered two fragments of rBet v1 (F1, aa 1–73 without methionine; F2, aa 74–159) and two trimers (comprising three covalently linked copies of Bet v 1) applied in eight doses (maximum dose of 80 µg) before the birch season. These recombinants induced the synthesis of IgG1 and IgG4 after treatment; despite a slight decrease, the antibodies remained present during the pollination season and decreased the release of histamine in serum and IgE levels [153]. Interestingly, an increase in IgG1, IgG2, and IgG4 was identified in the nasal secretion and is associated with reduced nasal sensitivity in the nasal birch challenge [154]. Additionally, the trimer of Bet v 1 decreased the production of the Th2 profile but increased the IL-12 levels, and both recombinant proteins decreased the nasal symptoms and skin reactivity [155].

Allergen-specific T lymphocytes (LT CLA^+^ and CCR4^+^, necessary for the migration of T cells from the blood to the skin) were found to increase after an epicutaneous injection of both rBet v1 and two fragments of this protein, in addition to a slight increase in IgG levels and its subclasses but a null humoral IgE response [156].

Other recombinants have been studied. For example, Meyer W. evaluated the response to the rBet v 1-folding variant, which has intact T cell epitopes, in a phase-III study. After exposure to AR patients for eight hours in an environmental exposure chamber with birch, the researcher applied a 10-dose injection scheme (20, 80, 160, and 320 µg) applied weekly, noting that the 80-µg dose of this recombinant induced the greatest synthesis of IgG1, reduced the nasal symptoms, and induced minimal adverse effects [157]. rBet v1 was also tested sublingually in a phase-II study, administering one sublingual tablet per day for five months before the pollination season; this treatment decreased the symptoms and use of rescue medications during the pollination season, with mild effects [158].

### 7.3. Grasses

From 1999, Gehlhar K. applied two recombinants (5a and 5b) with a homogeneity of approximately 70% with Phl p 5 in pediatric patients. These molecules decreased the AR symptoms and increased the levels of IgG, IgG2, and IgG4 at the end of the study; even the quotient IgG1/IgG4 correlated with the clinical scenario [159].

Recently, a fusion protein based on allergen-derived peptide B cell epitopes of the four major allergens of timothy (Phl p 1, 2, 5, and 6) and PreS protein (an immunogenic carrier that fosters antibody responses [160] from the hepatitis B virus—HBV), adsorbed to aluminum hydroxide, known as BM32, has been proven in patients with AR to grasses [161]. A two-year scheme was used to test BM32 in a phase-IIb study. In the first year, the researchers applied four injections; the initial three dosages were applied three months prior to the European grass pollen season and a booster in the fall (after the season) (Figure 1b). In the second year, they reapplied the first three doses of the scheme mentioned before the next pollination season. With this scheme, an increase in IgG, IgG1, and IgG4 was observed, but this effect declined after five months, particularly for IgG1. However, the booster was sufficient to restore the titers of IgG1 and increase the allergen-specific IgG4 levels. BM32 did not significantly modify the IgE levels compared with the baseline values. In terms of therapeutic advantages, phase-IIb studies showed benefic changes in AR life quality and asthma symptoms during the pollination, and these effects increased in the second year of treatment [162], with the main adverse reactions classified as mild [163].

Allergic mast cell and basophil degranulation may be prevented by the presence of blocking IgG1 and IgG4 antibodies against the IgE binding sites of the major grass pollen allergens. Likewise, as observed in phase II studies, blocking antibodies hinders the IgE-facilitated allergen presentation and the consecutive T cell activation [160]. In the same context, it inhibited the allergen-specific T cell reactivity in both treated patients and in vitro models [134]. Additionally, BM32 induces IL-10 synthesis and low levels of IL-5 and interleukins used as markers of immunological efficacy and tolerance [164].

## 8. Passive Immunization with IgG Antibodies

Passive immunization using serum from AIT-treated patients was first used by Cooke RA in 1935 [165]. In addition, the administration of IgG antibodies against parvalbumin decreased the allergic reactions in a mouse model of fish allergy [166]. A separate study was undertaken in a model of peanut allergy, where a treatment with allergen-specific IgG antibodies prevented peanut-sensitized mice from suffering anaphylaxis after the intravenous challenge with the whole peanut extract [167]. As previously reported, a clinical research in cat-allergy patients who were passively vaccinated with monoclonal IgG antibodies against the main cat allergen demonstrated an improvement in their symptoms [168].

## 9. Benefits and Limitations of Novel Immunotherapies

Strong benefits are associated with using hypoallergenic therapy, among which three are notable: preventing the development of allergies in sensitive patients, inducing immunity with allergens related to the primo-sensitizer, and conferring immunity to other entities. In the first case, based on a longitudinal study, the recognition of specific allergen-IgE in early life often precedes allergic symptoms or an allergic disease [169]. Campana R. described the effects of prophylactic AIT in non-allergic patients, but IgE-positive birch using recombinant rBet v1 (aa 1–160) and two hypoallergenic fragments of this protein (F1 aa 1–74 and F2 aa 75–160) in a scheme of three dosages applied monthly before the pollination season, and extra doses one year later, showed that the IgG concentrations increased in patients treated with the recombinants compared with the placebo group. Despite this change, IgG titers decreased at the end of the pollination season. However, they increased after a booster was administered before the following season. Interestingly, the patients who had received both rBetv1 and F1/F2 showed a decreased birch-specific IgE and no reactivity to the skin test, suggesting that IgG antibodies mediated the blockade of IgE. Additionally, the patients who had received active treatment showed no adverse reactions (Figure 1c) [170].

On the other hand, birch sensitivity is not related only to allergic respiratory diseases. Seventy percent of patients with food allergies (FAs) to apple and hazelnut develop sensitivity to this aeroallergen [171], and insufficient evidence exists that convectional AIT with extracts of birch improves the FA symptoms [172]. The administration of 80 µg of the rBet v 1 folding variant elevated IgG4 levels toward Bet v 1 and its related allergens contained in soja, apple, and cherry [173]. Kinaciyan T. described the effects of rMal d 1 and rBet v1 in patients with FA in a phase-II study using one sublingual tablet daily for 16 weeks (25 µg/day). When the patients were challenged through sublingual provocation with apple, the group that had received rMal d 1 showed fewer oral symptoms related to apple compared with rBet v 1 and enhanced IgG4 titers for apple but not rBet v 1. Instead, the patients who had received rBet v 1 induced a protective response to apple [174]. This latter finding has been shown in other allergens from fagal families [175].

Interestingly, after five applications, BM32 can also induce the synthesis of IgG, IgG1, and IgG4 against the N-terminal portion of PreS, whose ligand (sodium taurocholate cotransporter polypeptide located on the surface of hepatocytes) is necessary for the entry of the virus. These findings suggest that some components of AIT with recombinants can induce protective immunity against infectious diseases [176].

Although the clinical benefits of novel forms of AIT show promising results, some limitations should be addressed. For example, the use of hypoallergenic recombinant derivatives designed to reduce IgE reactivity [148] while maintaining T cell epitopes can still cause T-cell-mediated late-phase side effects. In this context, the employment of short non-allergenic peptides containing T cell epitopes that are not IgE-reactive can also cause adverse side effects in treated patients [132]. Additionally, T cell epitopes are too short to induce allergen-specific IgG antibodies and clinical protection [177].

Recently, a DNA vaccine was shown in phase Ia and Ib to treat a Japanese red cedar allergy employing the allergen CryJ2, and the lysosomal-associated membrane protein 1 (LAMP-1) induced humoral protective antibodies against this pollen [178]. This therapy may induce an uncontrolled synthesis of allergens in the body, causing allergic reactions [179]. A passive immunization with monoclonal IgG antibodies against the sensitizing allergen is a good approach. However, the high costs associated with large-scale antibody production remain a significant barrier [168]. The latest generation of hypoallergenic carrier-bound B-cell epitope-containing vaccines appears to be able to overcome the problem of the side effects. This allows for high-dose injections, induces robust allergen-specific IgG responses, does not cause allergic sensitization, and thus holds great promise for revolutionizing AIT and even for prophylactic allergy vaccination [162].

## 10. Recombinants for Diagnosis

Component-resolved diagnosis (CRD) was established in 1980 as a new concept in allergy diagnosis [180,181]. The CRD identifies a specific IgE toward purified natural or recombinant allergens rather than raw allergen extracts to determine a patient’s sensitization at the molecular level [182]; currently, more than 130 allergen molecules are commercialized [183]. CRD allows for a more precise identification of the allergen, using two types of tests for either one assay per sample (singleplex-ImmunoCAP, ImmuLite, and HyTech) or many allergens per sample depot in microarrays (multiplex platform- ImmunoCAP ISAC-ThermoFisher Scientific/Phadia) [184,185,186,187]. For example, in the case of patients allergic to several types of grass, these tests can help allergists distinguish between a major sensitization agent such as Phl p 1 and a pan allergen implicated in a crossover reaction with other species, such as Phl p 12 or Bet v1. This example can be applied to other proteins (profilins, polcalcins, non-specific lipid transportation proteins, PR-10, or tropomyosins). Recently, Armentia A. demonstrated the cross-reaction between different species, and marijuana and tomato can share lipid-transported proteins [185,188,189].

## 11. Allergy Proteomics

Over the last few years, proteomics has become critical to identify and structurally characterize allergens. Indeed, proteomics applications include in vitro diagnostics, allergen discovery, and the analysis of biologicals proposed for AIT [190,191]. Immunoproteomics involves a combination of proteomics with bidimensional polyacrylamide gel electrophoresis followed by mass spectrometry (MS) [189]. Briefly, the proteome of a biological specimen (e.g., pollen extract) is separated first by isoelectric focusing (first dimension) to separate proteins depending on their charge, and then by molecular masses (second dimension). Thus, hundreds of proteins are resolved as minute spots on a polyacrylamide gel, followed by western blotting to a membrane using sera from allergic patients. Then, IgE-reactive spots are characterized by MS. Although immunological methods have long been considered the gold standard for allergen identification, MS offers considerable benefits by allowing allergen identification based on MS/MS data from the allergen of interest [192]. For example, we have previously identified novel allergens from *Ligustrum lucidum* including enolase, pollen-specific polygalacturonases, Fra e 9.01 (β-1,3-glucanase), profilin, alanine aminotransferase, and ATP synthase beta subunit [175]. Using a similar approach, allergens from both red oak and pecan [189,191] have been recognized. The identification of enolase from some kinds of pollen is of particular interest [193]. Currently, proteomic technology is mostly used for research. Proteomic-based miniaturized technologies that allow for a more accurate, faster, and easier diagnosis of allergic sensitization are likely to contribute to the emergence of individualized AIT suited to individual allergic patients in the near future.

## 12. Conclusions

Despite the development of novel allergen-specific immunotherapy, licensing any vaccine for the clinic has proven difficult. Currently, allergen-specific immunotherapy with natural allergen extracts is the only viable disease-modifying treatment for allergic patients based on long-term symptom relief, and it can also prevent AR from progressing to asthma. However, caution should be taken because allergen injection can be associated with adverse reactions and because of the allergenicity of natural extracts. The side effects are usually harmless and, in rare cases, can cause fatal reactions. Importantly, patients must not show symptoms because of AIT allergenicity, particularly in asthma [194]. Traditional allergen extract-based AIT may be revolutionized in the future by some molecular AIT technologies. The latest generation of carrier-bound B-cell epitope-based allergy vaccines has the potential to transform AIT because it may prevent side effects, allowing the administration of high doses to induce strong allergen-specific IgG responses and providing sensitized patients with lasting effects (Supplementary Table S1).

## Figures and Tables

**Table 1:** Examples of new immunotherapy molecules.

Adjuvants	Hybrid Proteins	Recombinants
Aluminum	BTH2 (*Blomia tropicalis*)	MAT Fel d 1 (Cat)
Microcrystaline Tyrosine	DPx4 (*Dermatophagoides pteronyssinus*)	CatPAD (Cat)
Calcium Phosphate	MAVAC-BD-2 (*Blomia tropicalis* and *Dermatophagoides *sp.)	REGN1908 (Cat)
Toll-like Receptors		rBet v 1 FV (Birch)
Liposomes		rBet v 1 (Birch)
Virus Like Particles		BM32 (Grass)

## Data Availability

Not applicable.

## Supplementary Materials

The following are available online at https://www.mdpi.com/article/10.3390/cells11020212/s1, Table S1: Summary of allergoids, adjuvants and recombinants used in allergen immunotherapy.
