# Supplementary material for: Allergen Immunotherapy: Current and Future Trends
Source: Cells. 2022 Jan 8;11(2):212. doi: 10.3390/cells11020212 (PMC8774202; doi:10.3390/cells11020212)
Supplement: Supplementary file 1 [file cells-11-00212-s001.zip › cells-1452321-supplementary.pdf]

**Summary of allergoids, adjuvants, and recombinants used in allergen immunotherapy**					
**Type of molecule**	**Allergen**	**Brand**	**Clinical Phase**	**Efficacy**	**Reference**
Subcutaneous/ Sublingual Immunotherapy	Xolair	Omalizumab	IV	Omalizumab plus AIT reduced symptoms by about 40% compared to AIT exclusive. Likewise, the combination improved the quality of life of patients with rhinoconjunctivitis and asthma.	59
Allergoids	HDM	Acaroid ®	II	Significant reduction of the late-phase response and the use of inhaled corticosteroid especially with the 18,000 TU *vs.* placebo (69.2% *vs.* 20%; $p = 0.0160$).	68
				Reduction of IL-4, increase in IFN-γ levels, and improvement of perceived clinical conditions were evaluated by a VAS. Doses of 3,000 AU by week improve 78%/88% of the VAS at 6th and 12th months, respectively.	74
			IIb	Dosages at least 20,000 AUeq/mL ($p = 0.04$) or 50,000 ($p = 0.02$) AUeq/mL decrease the symptoms in the titrated nasal provocation test and increased the titters of IgG4 after 12 months of treatment.	67
	Grass	Grazax ®	III	27% of reduction of drugs during the pollination season *vs.* placebo, and 31% reduction symptoms in median rhinoconjunctivitis symptom score.	71
		Allergovit®	II	6,000 TU of *Phleum pratense* reduce 26% of the TNSSS score.	66
				The accelerated high-doses escalation (10,000 TU/mL) is safe and tolerable in comparison to the standard escalation schedule (1,000 TU/mL).	76
				The comparison between the accelerated dose-escalation scheme group and the conventional dose escalation scheme group showed that TEAEs (intensity of treatment-emergent adverse effect) were similar between the 2 schemes, 43.4% of patients only reported mild TEAEs.	77
			III	Increased the levels of allergen-specific IgG2 and IgG4 and showed improvement of 48% in symptom medication score after the second year *vs.* placebo.	65
			III b	The 6-grass allergic AIT applied pre/intra/post-seasonal is safe. The symptom and medication score improved significantly 48.4% after the second year of treatment.	64
	Birch	Allergovit®Birch	II	Both dosages of 1,000 and 10,000 TU/mL induce IgG2 and IgG4 titers.	69
		Purethal®	IV	20,000 AUM of Purenthal induces an increase of 2–5 times of the levels of IgG and IgG4 for birch and Bet v 1 like to conventional AIT.	70
Adjuvants	MCT	MATA-MPL	IV	AIT with MCT reduced the allergic symptoms −0.8(CI: −1.24, −0.36), the symptom score (−1.2 [CI: −2.11, −0.29]), and the total medication score (−2.2 [CI: −3.65, −0.74]).	97
	CaP			The scores of symptoms and medication score were significantly lower in the AIT group *vs.* placebo group (64.5 *vs.* 102.3). Augment the nasal reactivity threshold three times after AIT. AIT with CaP induced a reduction of cutaneous reactivity ($p < 0.001$). Likewise, augment 7% in the titers of IgG4.	100
	TLR	MPL®	I/II a	After ten weeks, patient groups that received SLIT with the highest MPL® developed the highest proportion of negative Grass allergen nasal challenge tests *vs.* placebo (47 and 44%, *vs.* 20%). Additionally, there were increased IgG levels and diminished IgE levels.	107
				Patients who completed the scheme recently (group 1), as well as those who completed it three years ago (group 2), had a reduction of five points in the weekly symptoms score. A great increase of IgG and IgG4 was observed in group 1, but the levels of group 2 were higher than placebo.	114
		AZD8848		Improved lung function in asthma patients. At 1 week after treatment, AZD8848 reduced the average late asthmatic response in FEV_1 by 27% compared to placebo. This effect lasted for four weeks after treatment.	117
	Liposomes			Mice treated with intranasal liposome-entrapped native Per a 9 had significantly lower inflammatory cells in the BALF in comparison to placebo (42.43 *vs.* 119.78 cells/microscopic field)	120
				Intranasal OVA-OML increased $CD4^+$ $CD25^+$ $Foxp3^+$ expression while suppressing allergic diarrhea.	121

				Immunization with a liposome-entrapped *D.pteronyssinus* extract diminished symptom and medication scores around 60% compared to placebo. Likewise, decreases the bronchial sensitivity during bronchial challenge by about 60%.	122
				The provocative cumulative dose of the allergen of Der p 1 need to induce a positive response in the bronchial challenge was 4 times higher in asthmatic patients treated with lyposme-entrapped *D. pteronyussinus* than the placebo group.	123
	VLP		IIb	The CYT003-QbG10 decreased 26% of total score of the Mini-RQLQ. Likewise, the conjunctival provocation test revealed a median 10-fold increase in allergen tolerance	126
				The average daily (asthma) symptom and medication score improved by 17% in patients treated with QbG10 *vs.* placebo.	127
Recombinants	Cat	CatPAD	II	The dose of 3 nmol of vaccine resulted in the inhibition of the late-phase skin response to intradermal whole allergen challenge (40% *vs.* 10%-placebo).	146
			III	Reduction of 3.85 units in TRSS units after 2 years.	147
			I	Intralymphathic recombinant increased 74-fold the nasal tolerance in comparison to placebo. In addition, it reduced skin reactions and increased the IgG4 and IL-10 levels.	149
			Ib	REGN1908 and REGN1909 block 51 % of Fel d 1 binding to IgE, interestingly, its combination has a blocking of 83%.	166
	Birch		II	Induced the synthesis of IgG mainly of allergen-specific IgG1, IgG2, and IgG4 after treatment and subtle induction of allergen-specific IgA and IgM.	151
			II	Tablets of rBet v 1 (12.5-50 mcg) decreased 17% of the symptoms during the pollination season, through ASS.	156
			III	A maintenance dose of 80 mcg of rBet v 1 FV reduces the total symptom score around 80% in comparison to placebo.	155
	Grass		IIb	BM32 increases 25% the quality of life during the pollen season in the first year and 31% in the second year.	160
			II	BM32 induced grass pollen allergen-specific IgG antibodies and HBV-neutralizing response.	158
Hybrid proteins design in silico	*Blomia tropicalis*		Pre clinical	BTH2 inhibited IgE-binding to rBlot 5 by 75.20% and inhibited IgE-binding to rBlot21 by 65.17%.	139
	Dermatophagoides pteronyssinus			IgE binding to *D. pteronyssinus* extract was inhibited by DPx4-specific IgG antibodies, which produced a high percentage of inhibition ranging from 39% to 78%.	140
	Dermatophagoides sp.			MAVAC-BD-2 reduced IgE reactivity to Der p 2 and Blo t 5 by 26%.	138

Supplementary Table S1. Summary of allergoids, adjuvants and recombinants used in allergen immunotherapy. AIT, Allergic-specific Immunotherapy; AAS, Adjusted Symptom Scores; BALF, Bronchoalveolar Lavage Fluid; CaP, Calcium Phosphate; CI, Confidence Interval; FEV_1, Forced Expiratory Volume in the first second; HBV, Hepatitis B Virus; HDM, House Dust Mite; IFN-γ, gamma interferon; Ig, Immunoglobulin; IL-4, Interleukin 4; IL-10, Interleukin 10; MCT, Microcrystalline Tyrosine; Mini-RQLQ, Mini Rhinoconjunctivitis Quality of Life Questionnaire; MPL®, monophosphoryl lipid A; OVA-OML, Ovalbumin-encased in oligomannose-coated liposomes; QbG10, Bacteriophage Qbeta-derived virus-like particle-containing CpG-motif G10; SLIT, Sublingual Immunotherapy; TEAEs, Treatment-emergent adverse events; TLR, Toll-like Receptor; ; TNSS, Total Nasal Symptom Severity Score; TRSS; Total Rhinitis Score; VAS, Visual Analogue Scale; VLP, Virus-like Particles.
